# Editorial

**DOI:** 10.2174/138920213804999165

**Published:** 2013-03

**Authors:** Christian Neri

As we are entering a new era of genomics and as sequencing and system-level approaches are boosting life science and human disease research, the journal Current Genomics will continue to provide the community with comprehensive reviews of last developments in the field. Current Genomics values openness, originality and high scientific standard and it will be the 2013 goal of the journal and its editorial board to keep working along these lines together with an expanding community of contributors and readers.

